# The Expression of miR-375 Is Associated with Carcinogenesis in Three Subtypes of Lung Cancer

**DOI:** 10.1371/journal.pone.0144187

**Published:** 2015-12-07

**Authors:** Yi Jin, Yalan Liu, Jin Zhang, Wei Huang, Hongni Jiang, Yingyong Hou, Chen Xu, Changwen Zhai, Xue Gao, Shuyang Wang, Ying Wu, Hongguang Zhu, Shaohua Lu

**Affiliations:** 1 Department of Pathology, School of Basic Medical Science, Fudan University, Shanghai, China; 2 Department of Pathology, the First Affiliated Hospital of Wenzhou Medical University, Wenzhou, China; 3 Department of Pathology, Zhongshan Hospital, Fudan University, Shanghai, China; 4 Key Laboratory of Molecular Medicine, Ministry of Education, Institute of Medical Sciences, Department of Biochemistry and Molecular Biology, Shanghai Medical College, Fudan University, Shanghai, China; 5 Department of Thoracic Surgery, Shanghai Pulmonary Hospital, School of Medicine, Tongji University, Shanghai, China; 6 Department of Pulmonary Medicine, Zhongshan Hospital, Fudan University, Shanghai, China; 7 Department of Pathology, Huashan Hospital, Fudan University, Shanghai, China; UCSF / VA Medical Center, UNITED STATES

## Abstract

Many studies demonstrated unique microRNA profiles in lung cancer. Nonetheless, the role and related signal pathways of miR-375 in lung cancer are largely unknown. Our study investigated relationships between carcinogenesis and miR-375 in adenocarcinoma, squamous cell carcinoma and small cell lung carcinoma to identify new molecular targets for treatment. We evaluated 723 microRNAs in microdissected cancerous cells and adjacent normal cells from 126 snap-frozen lung specimens using microarrays. We validated the expression profiles of miR-375 and its 22 putative target mRNAs in an independent cohort of 78 snap-frozen lung cancer tissues using quantitative reverse-transcriptase PCR. Moreover, we performed dual luciferase reporter assay and Western blot on 6 targeted genes (*FZD8*, *ITGA10*, *ITPKB*, *LRP5*, *PIAS1* and*RUNX1*) in small cell lung carcinoma cell line NCI-H82. We also detected the effect of miR-375 on cell proliferation in NCI-H82. We found that miR-375 expression was significantly up-regulated in adenocarcinoma and small cell lung carcinoma but down-regulated in squamous cell carcinoma. Among the 22 putative target genes, 11 showed significantly different expression levels in at least 2 of 3 pair-wise comparisons (adenocarcinoma vs. normal, squamous cell carcinoma vs. normal or small cell lung carcinoma vs. normal). Six targeted genes had strong negative correlation with the expression level of miR-375 in small cell lung carcinoma. Further investigation revealed that miR-375 directly targeted the 3’UTR of *ITPKB* mRNA and over-expression of miR-375 led to significantly decreased ITPKB protein level and promoted cell growth. Thus, our study demonstrates the differential expression profiles of miR-375 in 3 subtypes of lung carcinomas and finds thatmiR-375 directly targets *ITPKB* and promoted cell growth in SCLC cell line.

## Introduction

Lung cancer has long been the leading cause of cancer-related death in males worldwide[1]**.** Histologically, lung cancer is classified into 2 major classes, non-small cell lung cancer (NSCLC) and small cell lung cancer (SCLC). NSCLC is a heterogeneous group comprised of 2 most common subtypes, i.e. squamous cell carcinoma (SQ) and adenocarcinoma (AC)[2]**.**Despite the improvements in early diagnosis and recent breakthrough in chemo/targeted therapies, the overall 5-year survival rate of lung cancer remains low and the recurrence rate is high[3]**.**Poor prognosis is due to late disease presentation, heterogeneities, and relatively limited understanding of tumor biology. Therefore, discovery of new molecular markers and targets for the diagnosis and treatment of lung cancer would play pivotal roles in improving prognosis.

MicroRNA (miRNA) is a class of endogenously expressed, noncoding small RNA with around 22 nucleotides. It has been shown that miRNA scan regulate gene expression at the posttranscriptional level through imperfect base pairing with the 3’-untranslated region (3’UTR) of target mRNAs[4]**.** Growing evidence suggests that deregulation of miRNAs may contribute to certain cancer types including lung cancer**.** Many studies have demonstrated unique miRNA profiles in lung cancer [5–7].In our previous study on the investigation of miRNA biomarkers in 3 subtypes of lung carcinomas, we found significant up-regulation of the microRNA-375(miR-375) expression levels in AC and SCLC but down-regulation of miR-375 in SQ [8]. We hypothesize that miR-375 may be a candidate oncogene in AC and SCLC but a tumor suppressor in SQ. In fact, the phenomena of a single miRNA that plays opposite roles during tumor pathogenesis, either as an oncogene or as a tumor suppressor, has been reported in different cancers[9–11].In addition, up-regulation of miR-375 in SCLC was reported recently[12, 13]. However, signal pathways regulated by miR-375 and the role of miR-375 in lung cancer is still largely unknown.

We investigated the role of miR-375 in the carcinogenesis of 3 lung carcinoma subtypes to identify new molecular targets for diagnosis and therapy of lung cancer. In this paper, we show successful validation of distinct miR-375 expression profiles in the 3 subtypes. Furthermore, we present expression profiles of 22 putative target mRNAs of miR-375 in 3 lung carcer subtypes. In addition, we demonstrate that miR-375 promotes cell growth in SCLC cell line and inhibits ITPKB expression at the posttranscriptional level by directly targeting the 3’UTR of *ITPKB* mRNA.

## Materials and Methods

### Clinical specimens

The local Ethics Committee approved the study and written informed consent was obtained from all the patients. For sample selection, routine histological classification was used following World Health Organization Classification of lung tumors[2].The diagnosis of AC was confirmed by absence of keratinization or intercellular bridges and positive TTF1 staining. SQ diagnosis was confirmed by intercellular bridges or positive P63 staining (at least 40% of tumor cells). For all the SCLC cases, there was complete agreement on neuroendocrine morphology, small cells and neuroendocrine positive staining. If there was a disagreement, the consensus was reached by the detection of TTF1/P63. All cases were independently reviewed by 2 experienced lung cancer pathologists. Patients who had received pre-operative radiotherapy or chemotherapy were excluded. The clinical characteristics are presented in Table 1.

**Table 1:** Characteristics of patients and tumors in this study.

Variable	Microarrays					qRT-PCR			
	AC	SQ	SCLC	normal		AC	SQ	SCLC	normal
**Patients**	36	30	16			23	23	10	
**Tissue specimens**	36	30	16	44		23	23	10	22
**Sex**									
** Male**	13	26	14			8	22	9	
** Female**	23	4	2			15	1	1	
**Age (Mean±SD)**	58±10	62±10	58±10			61±10	64±9	59±8	
**TNM stage (NSCLC) **									
**Tumor stage (T) **									
**T1**	16	9			7		13		
**T2**	18	21			16		10		
**T3**	0	0			0		0		
**T4**	2	0			0		0		
**Nodule status (N)**									
**N0**	26	16			14		19		
**N1**	2	6			8		4		
**N2**	8	8			1		0		
**Distant metastases (M)**									
**M0**	36	30			23		23		
**M1**	0	0			0		0		
**Stage**									
**IA**	13	4			4		12		
**IB**	11	12			10		7		
**IIA**	1	1			3		1		
**IIB**	1	5			5		3		
**IIIA**	8	8			1		0		
**IIIB**	2	0			0		0		
**IV**	0	0			0		0		
**Stage (SCLC)**									
**Limited**			10					3	
**Extensive**			6					7	
**Differentiation**									
**Well**	3	3			0		0		
**Median**	16	7			20		14		
**Poor**	17	20			3		9		

### RNA isolation

Total RNA of the frozen tissue sections was extracted using mirVana miRNA isolation kit according to the manufacturer’s instructions (Applied Biosystems, Foster City, CA). The concentration was quantified by NanoDrop 1000 Spectrophotometer (NanoDrop Technologies, Waltham, MA). The quality control of RNA was performed by a 2100 Bioanalyzer using the RNA 6000 Pico LabChip kit (Agilent Technologies, Santa Clara, CA). The quality was measured using RNA integrity number (RIN). A RNA sample was discarded if the RIN score was less than 5.0.

### qRT-PCR

Quantitative reverse-transcriptase-polymerase-chain-reaction (qRT-PCR) was performed on 78 macrodissected frozen lung tissues to validate the expression profiles of miR-375 and its putative targeted genes in 3 subtypes of lung carcinomas. In the validation of miR-375, qRT-PCR was performed using Taqman microRNA assays (Applied Biosystems, Foster City, CA) according to the manufacturer’s instructions. The U47 small nuclear RNA was used as an endogenous control. All assays were carried out in triplicate.

For 22 predicted target genes of miR-375 (*ACSL3*, *CACNG2*, *CCDC6*, *CSNK2A1*, *FZD8*, *ITGA10*, *ITPKB*, *JAK2*, *JUND*, *LAMC1*, *LRP5*, *MAP3K5*, *NLK*, *PAK7*, *PDGFC*, *PDPK1*, *PIAS1*, *RUNX1*, *RYR2*, *SOCS5*, *SP1*and*WNT5A*), qRT-PCR using SYBR Green PCR Master Mix kit (Applied Biosystems, Foster City, CA) was performed according to the manufacturer’s instructions. *GAPDH* was used as an endogenous control. All assays were carried out in triplicate. Primer sequences are available upon request. Exclusion criteria for statistical analysis are as follows: 1) A target gene that showed cycle threshold (CT) values above 35 cycles in > 20% of the tested samples and 2) A sample that showed CT values above 35 cycles in > 20% of the tested genes.

### Target prediction and pathway analysis

The targets of miR-375 were predicted through the gateway miRecords (http://mirecords.biolead.org/). To increase the prediction accuracy, the genes that were predicted by at least 4 of 11 databases (Diana, microinspector, miranda, mirtarget2, mitarget, nbmirtar, pictar, pita, rna22, rnahybrid and targetscan) were selected as targets.

The KEGG database (http://www.genome.jp/kegg/tool/search_pathway.html) was used to map the predicted targets to lung cancer-associated pathways.

### Cells

A SCLC cell line NCI-H82 was kindly provided by Dr. Xueliang Zhu (Institute of Biochemistry and Cell Biology, Shanghai Institutes for Biological Sciences, Chinese Academy of Sciences, Shanghai, China)[13]. NCI-H82 stably transduced with miR-375-expressing (H82-miR-375) or empty (H82-NC) lentiviruses were maintained in RPMI-1640 with 10% fetal bovine serum (FBS). SV40-transformed embryonic kidney cell line 293T, obtained from the American Type Culture Collection, was maintained in DMEM with 10%FBS.

### Vector construction

Six putative targeted genes (*ITPKB*, *RUNX1*, *LRP5*, *PIAS1*, *FZD8* and *ITGA10*) that had strong negative correlation with miR-375expression level in SCLC were selected for functional studies. Of the 6 targeted genes, the 3’UTR of *ITPKB* mRNA contained 3 putative target sites for miR-375 (target site 1: seed 1667–1673, target site 2: seed 2463–2469 and target site 3: seed 2513–2519). Two different segments of the *ITPKB* 3’UTR, designated ITPKB-Δ1 (target site 1) and ITPKB-Δ2 (target site 2 and 3), were cloned, respectively.Schematic representation of the *ITPKB* 3’UTR and the putative binding sites for miR-375 is shown in S2 Fig.

To construct the miR-375 expression vector (pS-miR-375), a DNA fragment encoding miR-375 pre-miRNA (flanking upstream and downstream 30–50 nt) was amplified and inserted into the *Bam*HⅠ and *Hind*Ⅲ sites of expressing vector pSilencer4.1 CMV-puro. The miR-375-expressing lentiviral vector was constructed with Ubi-EGFP-MCS-IRES-Puromycin.

To construct wild-type 3’-UTR pGL3 reporters (pGL3-ITPKB-Δ1, pGL3-ITPKB-Δ2, pGL3-RUNX1, pGL3-LRP5, pGL3-PIAS1, pGL3-FZD8 and pGL3-ITGA10), 100 bp DNA fragments of the predicted targeted genes including the putative miR-375 binding sequences and XbaⅠrestriction sites were synthesized and cloned into the XbaⅠ site immediately downstream of the stop codon in the pGL3-promoter vector. Mutated 3’-UTR pGL3 reporters (pGL3-ITPKB-Δ1-mut1, pGL3-ITPKB-Δ2-mut2, pGL3-ITPKB-Δ2-mut3, pGL3-RUNX1-mut, pGL3-LRP5-mut, pGL3-PIAS1-mut, pGL3-FZD8-mut and pGL3-ITGA10-mut), which carried the mutated sequence in the complementary site for the seed region of miR-375, were generated based on wild-type 3’-UTR pGL3 reporters by site-specific mutagenesis. All cloned products were verified by sequencing. Primers for cloning miR-375 and oligonucleotides for target 3’UTR construction are presented in S1 Table.

### Luciferase reporter assay

293T cells were seeded in 96-well plate. The cells were co-transfected with 2 ng of an internal control vector pRL-renilla (Promega, Madison, WI), 40 ng of different 3’-UTR pGL3-promoter reporters, 160 ng of miR-375 expression vector (pS-miR-375) or empty control (pS-negative). Forty-eight hours after transfection, the firefly and renilla luciferase activities were assayed using Dual-Glo Luciferase assay system (Promega, Madison, WI). The luciferase activity of each sample was normalized by renilla luciferase activity. The normalized luciferase activity for the pS-negative was set as relative luciferase activity 1. All experiments were performed in triplicate and independently repeated3 times.

### Western blot

NCI-H82 cells were infected with miR-375-expressing (H82-miR-375) or empty (H82-NC) lentivirus at a MOI of 80. The infection efficiency was about 100% as assessed by microscopy of GFP fluorescence. The cells were harvested after 72 hours. Total protein of the cells or fresh tissues (2 AC, 2 SQ, 3 SCLC and 1adjacent normal tissue) were prepared using RIPA lysis buffer. Cell protein lysates were separated in 10% SDS-polyacrylamide gels, electrophoretically transferred to polyvinylidenedifluoride membranes (Roche, Pleasanton, CA), then detected with antibodies including anti-ITPKB (for cell line: ab171984, Abcam; for tissue: NBP1- 81589, Novus Biologicals), anti-RUNX1(ab54869, Abcam), anti-LRP5 (ab38311, Abcam), anti-PIAS1(ab77231, Abcam), anti-FZD8 (ab155650, Abcam), anti-ITGA10 (ab118099, Abcam), anti-GAPDH (Santa Cruz) and peroxidise-conjugated secondary antibodies (Santa Cruz). The intensity of protein fragments was quantified using Quantity One software and was normalized by GAPDH.

### CCK-8 assay

NCI-H82 cells were infected with miR-375-expressing (H82-miR-375) or empty (H82-NC) lentivirus as mentioned above. H82-miR-375 or H82-NC cells were seeded in 96-well plates. The cells were incubated for 24h, 48h, and 72h, respectively. CCK-8 reagent (DOJINDO) was added to each well 3h before the end of incubation. Optical density value (OD) of each sample was measured at a wavelength of 450nm. All experiments were performed in triplicate and independently repeated3 times.

### Statistical analysis

For microarray data, one-way analysis of variance (ANOVA) with Benjamini-Hochberg correction (p ≤ 0.001) was performed to determine differentially expressed miRNAs among normal, AC, SQ and SCLC tissues. Hierarchical clustering was performed with Pearson correlation using the differentially expressed miRNAs.

For qRT-PCR data, an unpaired, unequal variance *t*-test with Benjamini-Hochberg correction (p<0.05) was applied to miR-375 and differentially expressed targeted genes in the comparisons of AC vs. normal, SQ vs. normal and SCLC vs. normal tissue. The targeted genes with corrected p< 0.05 and fold expression change > 2 were selected as candidates for further functional studies. All p values were two-sided.

Additional methods including laser capture microdissection (LCM), macrodissection and microarray hybridization are described in S2 File.

## Results

### The miR-375 expression profiles in three subtypes of lung carcinomas

**Fig 1:**
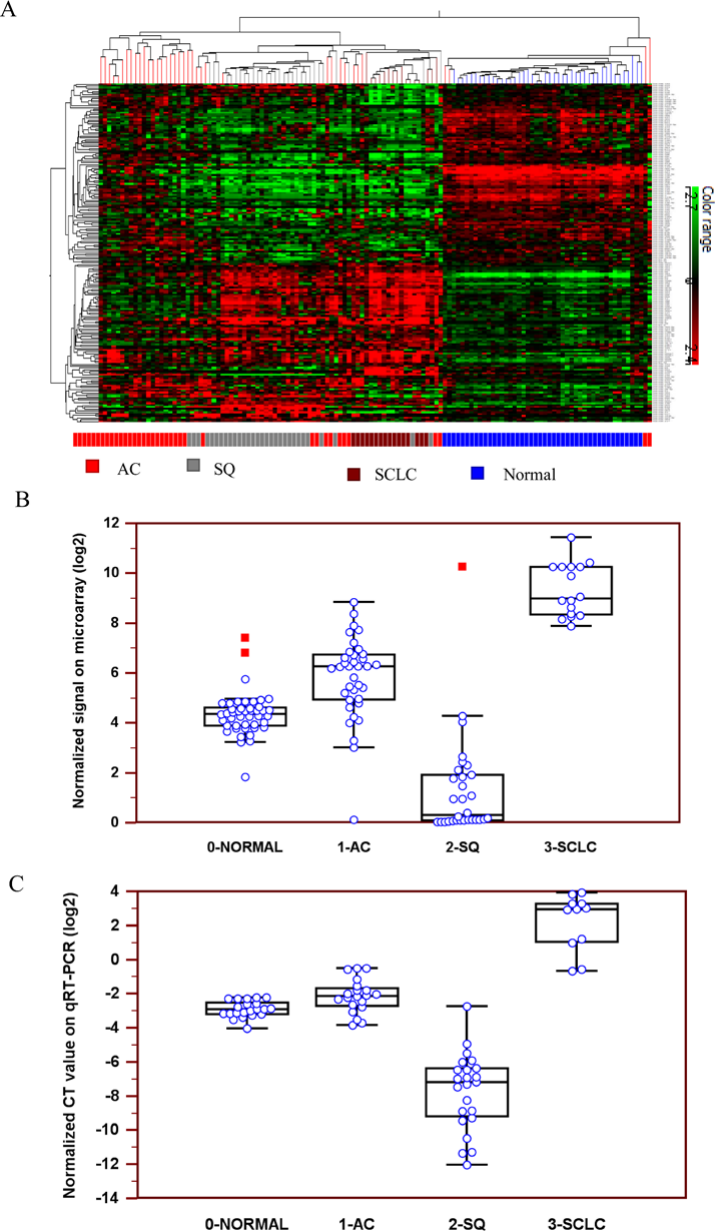
Expression profiles of miR-375 in 3 lung carcinoma subtypes. **A**, Hierarchical clustering of miRNA expression profiles in AC, SQ, SCLC and normal lung tissue on microarrays. The mean signal from biological replicate samples was used for the clustering. Colored bars indicate the range of normalized log2-based signals. **B**, An interactive dot diagram of miR-375 normalized signals on microarrays. **C**, An interactive dot diagram of miR-375 normalized CT values on qRT-PCR. AC: adenocarcinoma, SQ: squamous cell carcinoma, SCLC: small cell lung carcinoma.

In a microarray analysis, expression profiles of 723 human miRNAs were investigated in selected LCM cancerous cell populations and normal cells derived from 36 AC, 30 SQ, 16 SCLC and 44 adjacent normal tissues. Fig 1A illustrates the hierarchical clustering of the differential expression levels of miRNAs in 3 subtypes of lung carcinomas and normal lung tissue. The overall miRNA expression profiles clearly divided the samples into four major groups: AC, SQ, SCLC and normal tissue. Up-regulation of miR-375 expression level was observed slightly in AC and significantly in SCLC, while marked down-regulation of miR-375 expression was observed in SQ (Fig 1B and Table 2).

**Table 2:** Expression profiles of miR-375 in 3 subtypes of lung carcinomas.

	Microarrays		qRT-PCR	
	Corrected p-value	Fold change	Corrected p-value	Fold change
AC vs. normal	1.60E-05	2.8	4.30E-03	1.7
SQ vs. normal	6.70E-09	0.1	4.80E-10	0.04
SCLC vs. normal	2.60E-14	31.4	9.10E-07	34.2

The expression profiles of miR-375 in 3 lung carcinoma subtypes were further validated in an independent cohort of 78 snap-frozen surgical lung tissues using qRT-PCR. Similarly, miR-375 expression was significantly up-regulated in AC and SCLC but down-regulated in SQ (Fig 1C and Table 2).

### The association of miR-375 expression with lung carcinogenesis

We identified 191 genes through gateway miRecords to be associated with miR-375. After mapping to the KEGG pathway database, 22 genes were identified to be involved in important signaling pathways in lung cancer (S2 Table.).

The expression levels of the 22 putative target genes were next determined in 78 macrodissected frozen lung tissues. The expression profiles of the putative target mRNAs in 3 subtypes of lung carcinomas are presented in Table 3. Of the 22genes, 11 (*ACSL3*, *CACNG2*, *CCDC6*, *FZD8*, *ITGA10*, *ITPKB*, *JUND*, *LRP5*, *PIAS1*, *RUNX1*and*RYR2*) had markedly differential expression levels in at least 2 of 3 pair-wise comparisons of AC vs. normal, SQ vs. normal or SCLC vs. normal. Except *ACSL3*, all the 11 putative target genes showed significant down-regulation in all the 3 lung carcinomasubtypes. Conversely, *ACSL3* showed over 10 fold up-regulation when compareits expression level in SCLC to that in normal tissue (p = 0.011). Two genes (*CSNK2A1* and *MAP3K5*) showed differential expression levels in SQ vs. normal comparison only. Three genes (*NLK*, *PDPK1* and *SP1*) showed differential expression in SCLC vs. normal control only. Two genes (*JAK2* and *LAMC1*) showed comparable expression levels in the 3 comparisons, while other 4 genes (*PAK7*, *PDGFC*, *SOCS5* and *WNT5A*) did not pass the quality control in which the target gene showed CT values above 35 cycles in > 20% of the tested samples.

**Table 3:** Expression profiles of putative target mRNAs in miR-375.

ID	AC vs. normal		SQ vs. normal		SCLC vs. normal	
	Corrected p-value	Fold change	Corrected p-value	Fold change	Corrected p-value	Fold change
**ACSL3**	**3.6E-02**	**0.2**	**3.6E-02**	**0.2**	**1.1E-02**	**10.5**
**CACNG2**	**5.0E-03**	**0.2**	**3.3E-04**	**0.1**	6.9E-01	1.2
**CCDC6**	**1.9E-03**	**0.2**	**1.2E-08**	**0.1**	9.0E-01	0.9
**CSNK2A1**	8.7E-02	0.7	**2.5E-02**	**0.3**	4.9E-01	1.4
**FZD8**	**4.4E-03**	**0.2**	**1.3E-07**	**0.03**	**2.4E-02**	**0.1**
**ITGA10**	**6.0E-07**	**0.2**	**1.2E-12**	**0.01**	**9.1E-05**	**0.1**
**ITPKB**	**3.9E-04**	**0.1**	**1.2E-13**	**0.02**	**1.8E-04**	**0.04**
**JAK2**	8.0E-01	1.2	9.8E-01	1.0	6.8E-01	1.5
**JUND**	**3.9E-04**	**0.1**	**1.2E-08**	**0.1**	7.6E-01	0.8
**LAMC1**	8.2E-01	1.2	2.0E-01	0.6	5.6E-01	1.6
**LRP5**	**2.5E-03**	**0.2**	**3.0E-10**	**0.03**	**5.1E-04**	**0.2**
**MAP3K5**	3.1E-01	0.6	**3.0E-02**	**0.4**	6.6E-01	1.3
**NLK**	8.5E-01	0.9	6.5E-01	0.8	**3.2E-04**	**19.9**
**PAK7**	ND	ND	ND	ND	ND	ND
**PDGFC**	ND	ND	ND	ND	ND	ND
**PDPK1**	6.3E-01	1.6	6.1E-01	0.6	**4.0E-05**	**49.5**
**PIAS1**	**9.4E-06**	**0.1**	**1.0E-13**	**0.01**	**8.0E-05**	**0.2**
**RUNX1**	10.0E-01	1.0	**1.5E-11**	**0.1**	**2.0E-03**	**0.3**
**RYR2**	**1.2E-02**	**0.2**	**7.0E-06**	**0.1**	**8.0E-01**	1.2
**SOCS5**	ND	ND	ND	ND	ND	ND
**SP1**	4.1E-01	1.7	3.3E-01	1.7	**3.4E-06**	**24.1**
**WNT5A**	ND	ND	ND	ND	ND	ND

AC: adenocarcinoma, SQ: squamous carcinoma, SCLC: small celllungcarcinoma.

ND: not determined, the target gene did not pass the quality control process in which the target gene showed CT values above 35 cycles in > 20% of the tested samples.

### Correlation of miR-375 level with target mRNA expression

**Fig 2:**
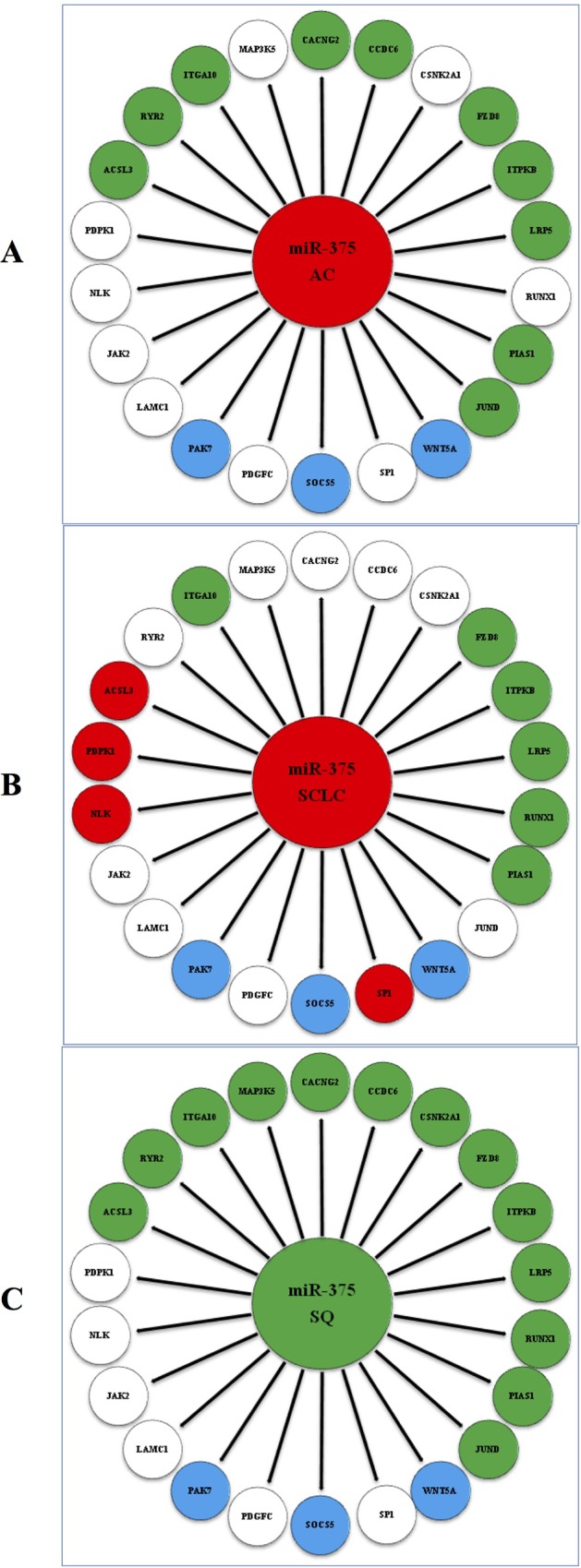
Correlation of miR-375 with target mRNA expression. **A,** Correlation of miR-375 with target mRNA expression in the comparison of AC vs. normal. **B,** Correlation of miR-375 with target mRNA expression in the comparison of SQ vs. normal. **C,** Correlation of miR-375 with target mRNA expression in the comparison of SCLC vs. normal. Green circle: down-regulation. Red circle: up-regulation. White circle: the gene did not show differential expression in the pair-wise comparison of AC vs. normal, SQ vs. normal or SCLC vs. normal. Blue circle: the gene did not pass the quality control. AC: adenocarcinoma, SQ: squamous cell carcinoma, SCLC: small cell lung carcinoma.

In the comparison of AC vs. normal (Fig 2A), strong negative correlations were observed between the expression level of miR-375 and that of 10 putative target mRNAs (*ACSL3*, *CACNG2*, *CCDC6*, *FZD8*, *ITGA10*, *ITPKB*, *JUND*, *LRP5*, *PIAS1*and*RYR2*). In the comparison of SQ vs. normal (Fig 2B), strong positive correlations were detected between the expression level of miR-375 and that of 13 putative target mRNAs (*ACSL3*, *CACNG2*, *CCDC6*, *CSNK2A1*, *FZD8*, *ITGA10*, *ITPKB*, *JUND*, *LRP5*, *MAP3K5*, *PIAS1*, *RUNX1* and *RYR2*). In the comparison of SCLC vs. normal (Fig 2C), strong negative correlations were found between the expression level of miR-375 and that of 6 putative target mRNAs (*FZD8*, *ITGA10*, *ITPKB*, *LRP5*, *PIAS1* and *RUNX1*), while strong positive correlations were observed between the expression of miR-375 and that of 4 putative target mRNAs (*ACSL3*, *NLK*, *PDPK1* and*SP1*).

### ITPKB: a direct target of miR-375

**Fig 3:**
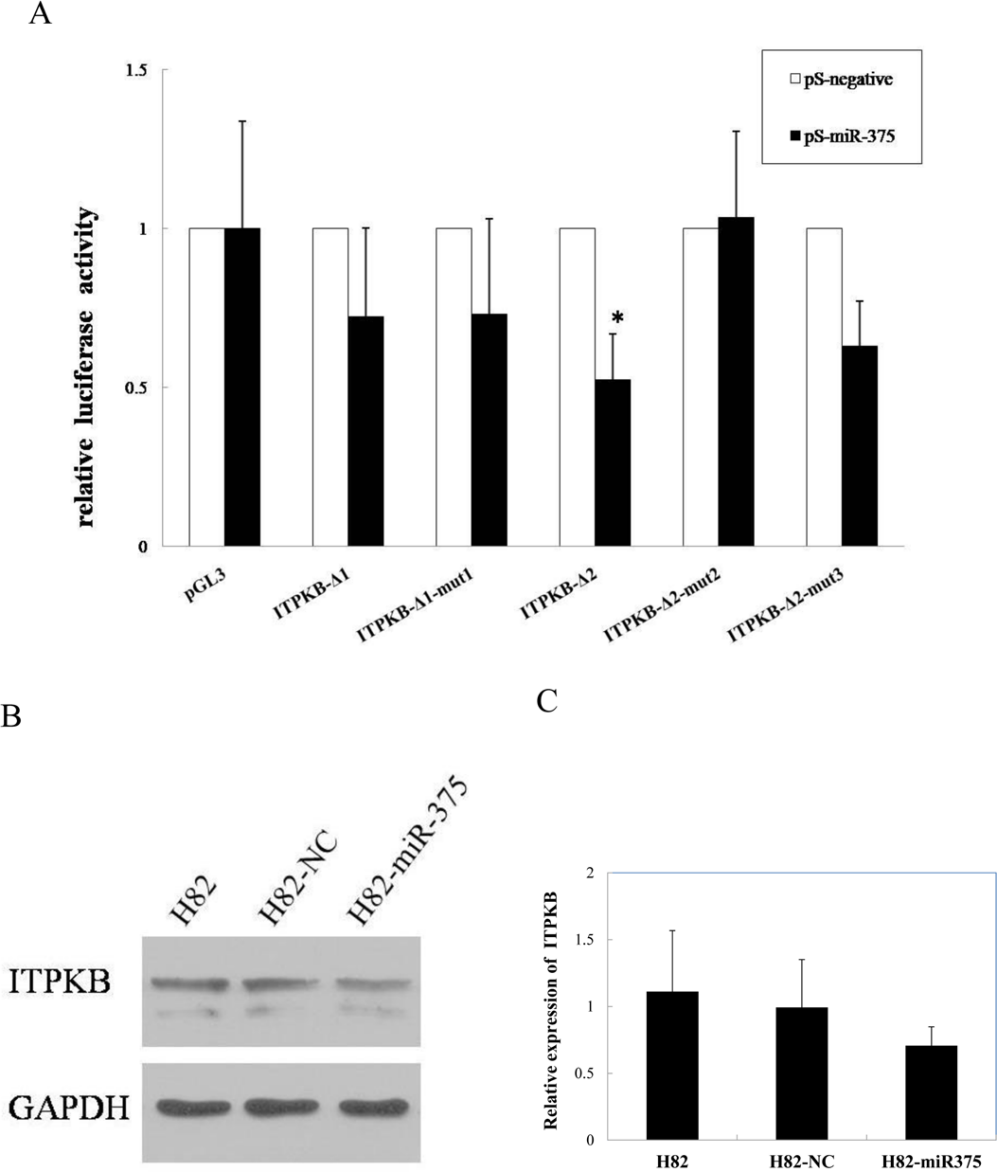
ITPKB: a direct target of miR-375. A**,** Luciferase activity.The luciferase activity of each sample was normalized by Renilla luciferase activity. The normalized luciferase activity for the pS-negative was set as relative luciferase activity 1. Column: mean value of 3 independent experiments in triplicate. Error bar: standard deviation. *: p< 0.05, compared with the cells transfected with empty pGL3-promoter vector. **B,** The expression levels of ITPKB protein of H82, H82-NC and H82-miR-375 cells were evaluated by western blot. After the transfection of miR-375, the expression level of ITPKB protein was significantly suppressed. C, Quantitative analysis of Western blot. The ITPKB protein intensities were normalized by GAPDH.

Dual luciferase reporter assay was performed on 6 targeted genes (*FZD8*, *ITGA10*, *ITPKB*, *LRP5*, *PIAS1* and*RUNX1*) that had strong negative correlation with the expression level of miR-375 in SCLC. For the 3 putative target sites at the 3’UTR of *ITPKB* mRNA, the luciferase activity of the reporter carrying the site 2 and 3 wild-type (ITPKB-Δ2) was significantly suppressed by miR-375, while the luciferase activity of the site 1 wild-type (ITPKB-Δ1) was unaffected. Importantly, the site 2 mutant (ITPKB-Δ2-mut2) was not targeted by miR-375, while the site 3 mutant (ITPKB-Δ2-mut3) was still inhibited by miR-375 (Fig 3A). Taken together, the results revealed that miR-375 targeted the 3’UTR of the *ITPKB* mRNA primarily through the target site 2 (seed: 2463–2469).

The effect of miR-375 on the endogenous expression of ITPKB was further examined by Western blot. The ectopic expression of miR-375 significantly suppressed the ITPKB protein in the miR-375-transfected H82 cells (H82-miR-375) compared to the vector control transfected H82 cells (H82-NC) (Fig 3B and 3C). The data indicated that miR-375 inhibited the expression of ITPKB at the posttranscriptional level by directly targeting the 3’UTR (primarily target site2) of *ITPKB* mRNA.

**Fig 4:**
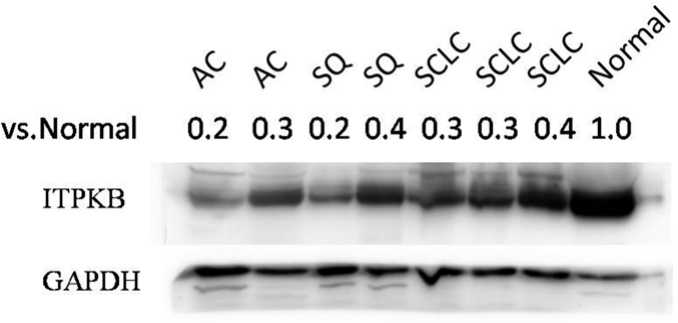
Expression of ITPKB protein in 3 lung carcinoma subtypes. Western blot was used to detect the expression of ITPKB protein in lung carcinoma tissues (2AC samples, 2SQ samples, 3SCLC samples) and adjacent normal lung tissue. The ITPKB protein intensities were normalized by GAPDH.The fold changes were 0.2and0.3 in the comparisons of 2AC vs. normal, 0.2 and 0.4of 2SQ vs. normal, 0.3, 0.3 and 0.4of 3SCLC vs. normal, respectively.

In addition, we observed ITPKB protein expression was significantly down-regulated in lung cancer tissues compared with adjacent normal tissue, which are in consonance with the expression pattern of *ITPKB* mRNA mentioned above (Fig 4 and Table 3).

For other 5 putative targeted genes (*RUNX1*, *LRP5*, *PIAS1*, *FZD8* and *ITGA10*), minimal effects on miR-375 were observed in their luciferase activities (S3 Fig). Furthermore, the ectopic expression levels of miR-375 were not significantly suppressed by the 5 putative target proteins in H82-miR-375 compared to H82-NC (S4 Fig).

### Effect of miR-375 overexpression on SCLC cell growth

**Fig 5:**
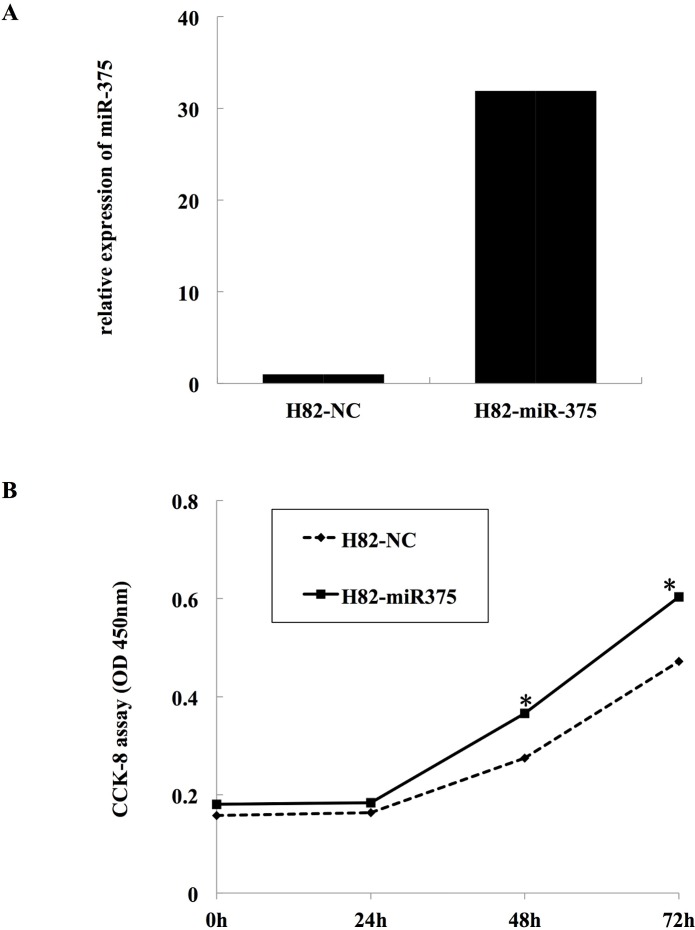
Effect of miR-375 on cell growth of NCI-H82. **A,** miR-375 expression of both H82-NC and H82-miR-375.NCI-H82 cells were infected with miR-375-expressing (H82-miR-375) or empty (H82-NC) lentivirus prior to the proliferation assay. **B,** miR-375 could promote cell growth of H82 cells. The infected cells were seeded to 96-wells plates. After incubation for several days, the cell growth was measured by CCK-8 assay. *: p< 0.01, compared with H82-NC.

The significant up-regulation of miR-375 expression in SCLC samples and its inhibitory effect on *ITPKB* prompted us to investigate its possible biological role in SCLC cells. After infected, miR-375 expression of both H82-NC and H82-miR-375 was detected using real-time qRT-PCR (Fig 5A). Then we evaluated the cell growth rate by CCK-8 assay. The results shown in Fig 5B suggested that exogenous expression of miR-375 could promote cell growth of H82 cells in a time-dependence manner. After 24h incubation, no difference in cell viability between the two groups was observed(p>0.05).However, significant difference between the two groups was observed after 48h and 72h incubation, and the promotion efficiencies were 33.1% and 35.9%, respectively(p<0.01).

## Discussion

MiR-375 was first identified as a pancreatic islet-specific miRNA regulating insulin secretion [14–16]. However, further studies revealed that miR-375 is a multifunctional miRNA participating in pancreatic islet development, glucose homeostasis, mucosal immunity, lung surfactant secretion and more importantly, tumorigenesis. Deregulation of miR-375 has been observed in different types of cancers. For instance, Kong et al reported that miR-375 expression was strongly downregulated in oesophageal squamous cell carcinoma (ESCC), and miR-375 inhibited tumor growth and metastasis through repressing insulin-like growth factor 1 receptor (IGF1R)[17]. Another study convinced that miR-375 was frequently downregulated in ESCC cell lines and tissues, and miR-375 was significantly associated with advanced stage, metastasis and poor outcome of ESCC [18]. Leidner et al found that miR-375 was associated with the progression to invasive carcinoma in Barrett’s esophagus [19].Chang et al showed that downregulation of miR-375 in glioma was correlated with unfavorable clinical outcome [20]. Low expression of miR-375 was found in the colorectal cancer [21, 22], and also correlated with poor outcome and metastasis in head and neck squamous cell carcinomas [23, 24]. He et al found that miR-375, downregulated in hepatocellular carcinoma (HCC) tissues and cell lines, targets AEG-1 in HCC and suppresses cancer cell growth *in vitro* and *in vivo*[25]. In lung cancer, decreased miR-375 expression level in NSCLC tissue samples significantly correlated with advanced stage and lymphatic metastasis[26].Reduced expression of miR-375 was observed in all cancer types mentioned above, while miR-375 upregulation was noticed in breast cancer and prostate carcinoma [27, 28]. These findings emphasize a fundamental role of miR-375 in tumorigenesis. Our results were consistent with previous findings. We discovered and validated significant miR-375 up-regulation in AC and SCLC but significant down-regulation in SQ.

The current two reports on miR-375 expression in SCLC were consistent with our results [12, 13]. Compared to them, our study is unique for the following reasons: First, Zhao et al examined miRNA expression patterns only in cell lines (four SCLC cell lines (H209, H69, H82, and H345), one NSCLC cell line CRL 5908 and one immortalized human lung epithelial cell line Beas-2B), they found that miR-375 was consistently upregulated in all four SCLC cell lines. The other one also drew the conclusion by cultured cells, and used small samples of tissue (7 NSCLC vs. 8SCLC) for verification without normal control. Our study not only included cell lines but also a more comprehensive scanning of clinical samples, enabling us to better identify potential diagnostic miRNA markers. Underscoring the miRNA expression profiles in subtyping SCLC and NSCLC is due to the difficulties in obtaining primary tissue specimens, as most SCLC tumors are not surgically resected. Second, we discovered miRNA biomarkers on LCM selected cancer cells and adjacent normal cells derived from AC, SQ and SCLC. This approach assures the purity of target cells, reducing background signals from non-cancerous cells and improving the reliability of the discovered biomarker candidates[29].And interestingly, we observed that overexpression of miR-375 can drastically promote cell growth in SCLC cell line NCI-H82.

MiRNAs are negative post-transcriptional regulatory elements of gene expression. Single miRNA can regulate multiple mRNAs and are possibly better drug targets. To better understand the molecular mechanism of miR-375 in the 3 subtypes of lung cancer, we further predicted the target genes of miR-375 and filtered the putative target genes through KEGG pathways. We observed that22 target genes are the key members of established signalling pathways in lung cancer including calcium, insulin, Jak-STAT, MAPK, mTOR, PPAR, TGF-beta and Wnt pathways. Among these genes, we found that 6 target genes (*FZD8*, *ITGA10*, *ITPKB*, *LRP5*, *PIAS1* and*RUNX1*) had strong negative correlation with miR-375 expression level in the comparison of SCLC vs. normal tissue. The association between lung cancer and 4 target genes (*FZD8*, *LRP5*, *PIAS1* and*RUNX1*) has been previously reported [30–33].It has been reported that *FZD8*is highly expressed in A549 cell line and the dysregulation of *FZD8* might play key roles in human cancer through activation of beta-catenin-TCF pathway [30]. Lee et al demonstrated a significant down-expression of *LRP5* gene in 6 of 17 lung squamous carcinoma samples [31]. In H1299 cell line, *PIAS1* inhibited STAT1-mediated gene activation and the DNA binding activity [32]. In NSCLC patients, *RUNX* was more frequently methylated in tumor tissues than in noncancerous tissues [33].However, the functions of *ITGA10* and *ITPKB* in lung cancer are still unknown. In our study, *ITPKB* mRNA and protein expression was significantly down-regulated in lung cancer tissues. The luciferase assay revealed that *ITPKB* is the direct target gene of miR-375. The over-expression of miR-375 led to significantly decreased ITPKB protein level. The results confirmed that miR-375 negatively regulated *ITPKB* at the translation level. For *ITGA10*gene, we did not observe any effects on miR-375 in the luciferase activity and the suppression of the ITGA10 protein expression by miR-375.

Recently, miR-375 has been found to suppress core hallmarks of cancer by targeting several important genes like *AEG-1*, *YAP1*, *IGF1R* and *PDK1* [16].The target genes regulated by miR-375 may function spatiotemporally or in cooperation with each other in different cellular processes. Our identification of ITPKB gene as a direct target of miR-375 provides new insights into the mechanisms underlying tumorigenesis. ITPKB, a member of [Ins*P*3] 3-kinases family, was mapped to the telomeric end of human chromosome 1 and associated with calcium signaling pathway. It has been identified as a candidate gene in the pathogenesis of immune disorders, multiple sclerosis, Alzheimer disease, and malignant melanoma [34, 35]. Since little work has been reported on *ITPKB* function in lung cancer pathogenesis, further studies *in vitro* and *in vivo* are needed to explore the potential effect of *ITPKB* in lung cancer, which will be helpful for understanding the role of miR-375 in lung cancer and if miR-375 upregulation is a cause or effect of cancer pathogenesis. It is well accepted that the expression levels of miRNAs and their direct mRNA targets may be negatively correlated[36, 37].Intriguingly, we did observe significant positive correlation in the expression level of miR-375 and its 13 putative targets in SQ and its 4 putative targets in SCLC. It has been demonstrated that a miRNA can function as a switch from repression to activation of its target genes [38, 39].Further functional studies are needed to investigate the role of the putative targets showing positive correlation with miR-375 expression level.

In summary, we discovered and validated thatmiR-375 expression level is significantly up-regulated in AC and SCLC but down-regulated in SQ. Furthermore, we found that miR-375 directly downregulates *ITPKB* in SCLC and promotes cell growth in SCLC cell line, which were not performed in previous studies. This study may be a first step. Further studies in the mechanisms of pathogenesis are needed to explore the potential clinical value of miR-375.

## Supporting Information

S1 Fig Laser capture microdissection of lung squamous cell carcinoma. A, H&E-stained slide (X 20). B, Hematoxylin stained slide before LCM (X 20). C, Hematoxylin stained slide after LCM (X 20). D, Cap showing adherent cells (X 20).
(TIF)

S2 Fig Schematic representation of the ITPKB 3’UTR and putative binding sites for miR-375. The 3’UTR of the ITPKB mRNA contains 3 putative target sites for miR-375 (site1: seed 1667–1673, site 2: seed2463-2469, site 3: seed2513-2519). Two different segments of the ITPKB 3’UTR, designated ITPKB-Δ1 (target site 1) and ITPKB-Δ2 (target site 2 and 3), were cloned, respectively. The mutation was generated in the complementary site for the seed region of miR-375, as indicated.
(TIF)

S3 Fig Luciferase assay of 5 putative target genes for miR-375. A, RUNX1. B, LRP5.C, PIAS1. D, FZD8. E, ITGA10.
(TIF)

S4 Fig Western blot analysis of 5 putative target genes. **A,** RUNX. **B,** LRP5. **C,** PIAS1. **D,** FZD8. **E,** ITGA10.
(TIF)

S1 File Abbreviations. (DOCX)

S2 File Supplementary materials and methods. (DOCX)

S1 Table Primers for cloning miR-375 and oligonucleotides for target 3’UTR construction (DOC)

S2 Table Putative targets of miR-375 in lung cancer signaling pathways. (DOC)
