# Supplementary figures and images for: The Expression of miR-375 Is Associated with Carcinogenesis in Three Subtypes of Lung Cancer
Source: PLoS One. 2015 Dec 7;10(12):e0144187. doi: 10.1371/journal.pone.0144187 (PMC4671676; doi:10.1371/journal.pone.0144187)

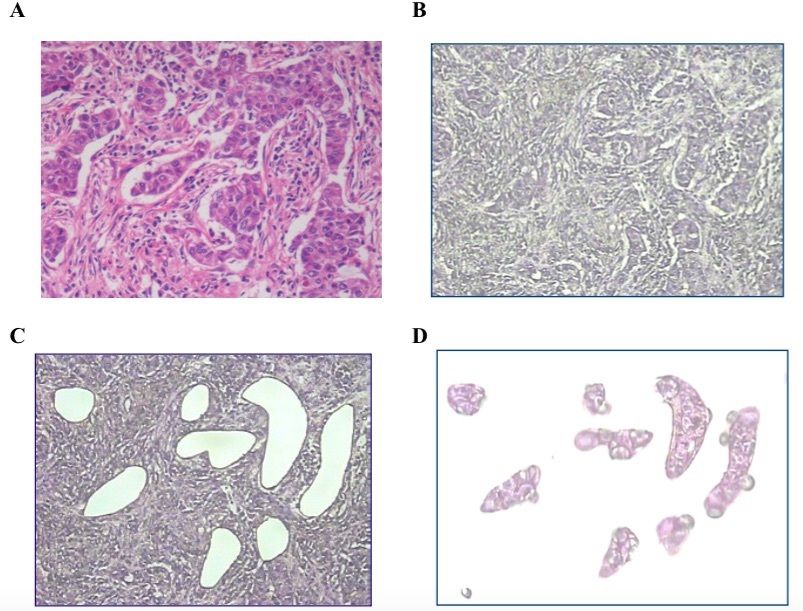

Supplement: S1 Fig — A, H&E-stained slide (X 20). B, Hematoxylin stained slide before LCM (X 20). C, Hematoxylin stained slide after LCM (X 20). D, Cap showing adherent cells (X 20). (TIF) [file pone.0144187.s001.tif]

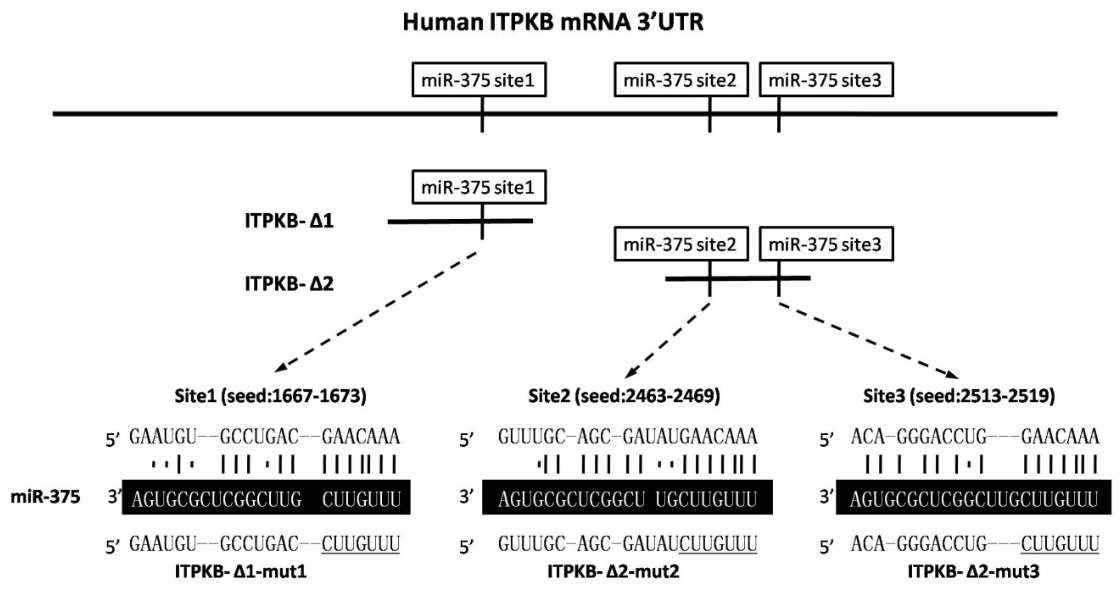

Supplement: S2 Fig — The 3’UTR of the ITPKB mRNA contains 3 putative target sites for miR-375 (site1: seed 1667–1673, site 2: seed2463-2469, site 3: seed2513-2519). Two different segments of the ITPKB 3’UTR, designated ITPKB-Δ1 (target site 1) and ITPKB-Δ2 (target site 2 and 3), were cloned, respectively. The mutation was generated in the complementary site for the seed region of miR-375, as indicated. (TIF) [file pone.0144187.s002.tif]

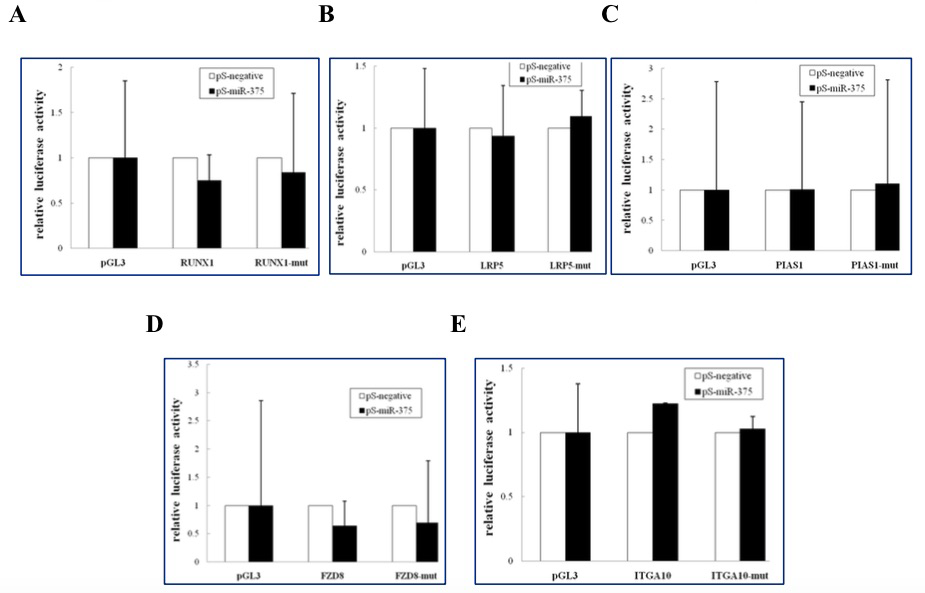

Supplement: S3 Fig — A, RUNX1. B, LRP5.C, PIAS1. D, FZD8. E, ITGA10. (TIF) [file pone.0144187.s003.tif]

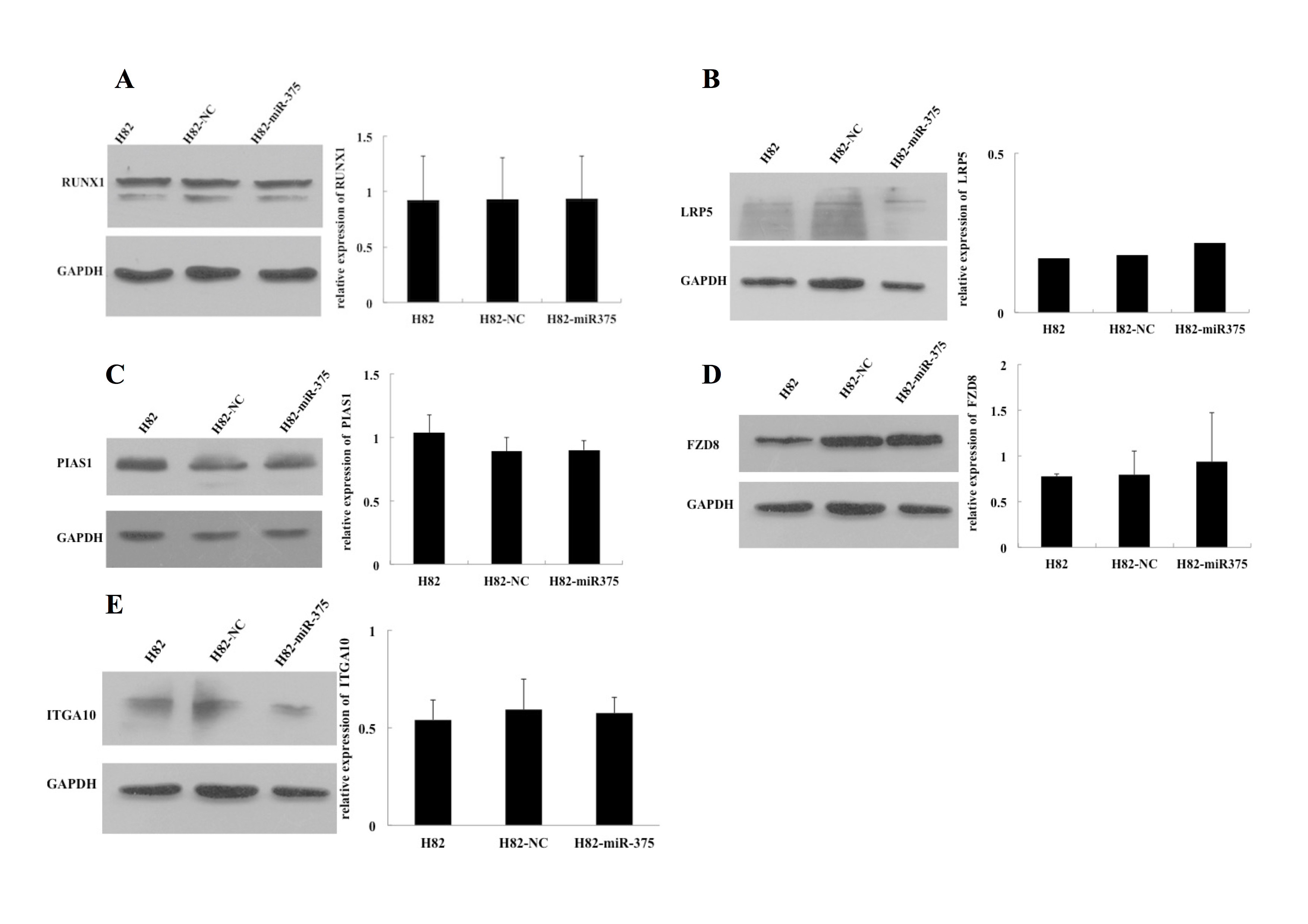

Supplement: S4 Fig — A, RUNX. B, LRP5. C, PIAS1. D, FZD8. E, ITGA10. (TIF) [file pone.0144187.s004.tif]
