# Supplementary material for: The Expression of miR-375 Is Associated with Carcinogenesis in Three Subtypes of Lung Cancer
Source: PLoS One. 2015 Dec 7;10(12):e0144187. doi: 10.1371/journal.pone.0144187 (PMC4671676; doi:10.1371/journal.pone.0144187)
Supplement: S1 File — (DOCX) [file pone.0144187.s005.docx]

**Abbreviations**

| **Abbreviations** | **Full name** |
| --- | --- |
| miRNA | microRNA |
| miR-375 | microRNA-375 |
| NSCLC | non-small cell lung cancer |
| SCLC | small cell lung cancer |
| SQ | squamous cell carcinoma |
| AC | adenocarcinoma |
| 3’UTR | 3’-untranslated region |
| RIN | RNA integrity number |
| qRT-PCR | Quantitative reverse-transcriptase-polymerase-chain-reaction |
| FBS | fetal bovine serum |
| LCM | laser capture microdissection |
| ESCC | oesophageal squamous cell carcinoma |
| IGF1R | insulin-like growth factor 1 receptor |
| HCC | hepatocellular carcinoma |
