# Supplementary material for: The Expression of miR-375 Is Associated with Carcinogenesis in Three Subtypes of Lung Cancer
Source: PLoS One. 2015 Dec 7;10(12):e0144187. doi: 10.1371/journal.pone.0144187 (PMC4671676; doi:10.1371/journal.pone.0144187)
Supplement: S2 File — (DOCX) [file pone.0144187.s006.docx]

**Supplementary materials and methods**

**Laser capture microdissection**

Tissue preparations on snap-frozen surgical specimens for laser capture microdissection(LCM) were performed as previously described. The target cell populations (~200,000 cells)were selected and captured using ultraviolet laser cutting following the manufacturer’s recommended protocol. Additionally, haematoxylin eosin(H&E)-stained sections were prepared for each tissue to guide the area of interest for LCM. Examples of histological images and LCM isolated cells from lung squemous cell carcinoma are shown in S1 Fig.

**Macrodissection**

H&E-stained sections on each tissue block were prepared to check the proportion of tumor material. If a tumor had more than 75% neoplastic cells, it was deemed suitable for analysis without further purification of tumor cells. If, however, histology showed the tumor having <75% neoplastic cells, it was selected and marked tumor for manual macrodissection.

**Microarray hybridization**

Human microRNA microarrays from Agilent Technologies were used in the discovery study. The microarray contains probes for 723 human microRNAs from Sanger database v.10.1. Each slide was formatted with 8 identical arrays. Total RNA (100 ng) derived from LCM-selected cells were labelled with Cy3. Microarray slides were scanned by XDR Scan (PMT100, PMT5). The labelling and hybridization were performed according to the protocols in Agilent microRNA microarray system. The microarray image information was converted into spot intensity values using Feature Extraction Software Rev. 9.5.3 (Agilent Technologies, Santa Clara, CA). The data obtained on a microarray were discarded for further analysis if the intra-array coefficient of variation across 16 replicated spots was higher than 15%.
