# Supplementary material for: The Expression of miR-375 Is Associated with Carcinogenesis in Three Subtypes of Lung Cancer
Source: PLoS One. 2015 Dec 7;10(12):e0144187. doi: 10.1371/journal.pone.0144187 (PMC4671676; doi:10.1371/journal.pone.0144187)
Supplement: S1 Table — (DOC) [file pone.0144187.s007.doc]

**S1 Table. Primers for cloning miR-375 and oligonucleotides for target 3’UTR construction**

| **Primer name** | **Sequence** (5' to 3', restriction sites are capitalized) |
| --- | --- |
| miR-375-F | gcgGGATCCagaccaggaccaggagatca |
| miR-375-R | gcgAAGCTTcccgtattacgacgcagaat |
|  |  |
| **Oligonucleotides** |  |
| ITPKB-Δ1 | CTAGAgccagtgagtctgatatggaagggaatgtaaaatttgcctgacttcttaagaacaaaacccccagctctgtgccccatgctccttggggcttgccacccaT |
| ITPKB-Δ1-mut1 | CTAGAgccagtgagtctgatatggaagggaatgtaaaatttgcctgacttcttaacttgtttacccccagctctgtgccccatgctccttggggcttgccacccaT |
| ITPKB-Δ2 | CTAGAgtgaaagaagtttgctaaagcaaatcatgatatgaacaaaaattacaggggacctgtttaagagaacaaaatgttccaagcactttaggcagacaccagcT |
| ITPKB-Δ2-mut2 | CTAGAgtgaaagaagtttgctaaagcaaatcatgatatcttgtttaattacaggggacctgtttaagagaacaaaatgttccaagcactttaggcagacaccagcT |
| ITPKB-Δ2-mut3 | CTAGAgtgaaagaagtttgctaaagcaaatcatgatatgaacaaaaattacaggggacctgtttaagacttgtttatgttccaagcactttaggcagacaccagcT |
| RUNX1 | CTAGAacatgaaaatttgtttgacaataatctcacaaaacatattttacatctgaacaaaatgcctttttgtttaccgtagcgtatacatttgttttgggattttT |
| RUNX1 -mut | CTAGAacatgaaaatttgtttgacaataatctcacaaaacatattttacatctcttgtttatgcctttttgtttaccgtagcgtatacatttgttttgggattttT |
| LRP5 | CTAGAcgggccactctggcttctctgtgcccctgtaaatagttttaaatatgaacaaagaaaaaaatatattttatgatttaaaaaataaatataattgggatttT |
| LRP5 -mut | CTAGAcgggccactctggcttctctgtgcccctgtaaatagttttaaatatcttgtttgaaaaaaatatattttatgatttaaaaaataaatataattgggatttT |
| PIAS1 | CTAGAcgtgttttttttcctttttttagggaaaaaattaaaagaaatgtacagagaacaaaactatattttcagttttacttttgtatataaatctaagactgccT |
| PIAS1 -mut | CTAGAcgtgttttttttcctttttttagggaaaaaattaaaagaaatgtacagacttgtttactatattttcagttttacttttgtatataaatctaagactgccT |
| FZD8 | CTAGAtcttaatggtatccattagctgggacttaaatgactcacttagaacaaagtacctggcattgaagcctcccagacccagccccttttcctccattgatgtT |
| FZD8 -mut | CTAGAtcttaatggtatccattagctgggacttaaatgactcacttacttgtttgtacctggcattgaagcctcccagacccagccccttttcctccattgatgtT |
| ITGA10 | CTAGAtcatgggcattgtctctgtttcccagtggggtggacagtatatcagatggtcagaacaaataaagttcagtgtcaaatgaaaaaaaaaaaaaaaaaaaaT |
| ITGA10-mut | CTAGAtcatgggcattgtctctgtttcccagtggggtggacagtatatcagatggtcacttgttttaaagttcagtgtcaaatgaaaaaaaaaaaaaaaaaaaaT |
