# Supplementary material for: The Expression of miR-375 Is Associated with Carcinogenesis in Three Subtypes of Lung Cancer
Source: PLoS One. 2015 Dec 7;10(12):e0144187. doi: 10.1371/journal.pone.0144187 (PMC4671676; doi:10.1371/journal.pone.0144187)
Supplement: S2 Table — (DOC) [file pone.0144187.s008.doc]

**S2 Table. Putative targets of miR-375 in lung cancer signaling pathways.**

| **Putative target** | **Pathway** |
| --- | --- |
| *ITPKB, RYR2* | Calcium signaling pathway |
| *PDGFC* | Cytokine-cytokine receptor interaction |
| *ITGA10, LAMC1* | ECM-receptor interaction |
| *ITGA10, LAMC1, PAK7, PDGFC, PDPK1* | Focal adhesion |
| *PDPK1* | Insulin signaling pathway |
| *JAK2, PIAS1, SOCS5* | Jak-STAT signaling pathway |
| *CACNG2, JUND, MAP3K5, NLK* | MAPK signaling pathway |
| *PDPK1* | mTOR signaling pathway |
| *CCDC6, RUNX1* | Pathways in cancer |
| *ACSL3, PDPK1* | PPAR signaling pathway |
| *SP1* | TGF-beta signaling pathway |
| *CSNK2A1, FZD8, LRP5, NLK, WNT5A* | Wnt signaling pathway |
